# Disease and Accident

**Published:** 1919-07-26

**Authors:** 


### DISEASE AND ACCIDENT.

DEATH due to disease differs widely from death due to other injury in many obvious respects. The actual occurrence and onset of the illness cannot be stated with the same certainty. The possibility of infection from other sources than the course of infection at present at work cannot be overlooked; and the difficulty of bringing these conditions within the common meaning of the term "accident" is in itself considerable.

The earliest case in which infection by hostile micro-organisms was held to be within the statute was *Britons, Ltd. v. Turvey* ([1905] A.C. 230). In that case a wool-sorter died of anthrax, and it was held that his representatives were entitled to recover. It was found as a fact by the County Court Judge who awarded compensation that the disease was caused by the accidental alighting of the bacillus from the infected wool on a part of the deceased person, which afforded a harbour in which it could multiply and grow, and so cause a malignant disease and consequent death. It was held that this was an accident, because, in the words of Lord Macnaghten, it was an accident that the noxious germ happened to be present in the material that the deceased was sorting, that it had escaped preventives provided by downdraught or suck of the fan, that it struck the man in the corner of his eye, and so found entrance into his system, and as this accident caused death the case was clearly within the statute. The importance of this decision lies in the fact that it included disease within the definition of accident, and disregarded the cases that had formerly decided that the onset of the disease must be the sequel of an accident causing physical injury received in the employment.

In *Elke v. Hart-Dyke* ([1910] 2 K.B. 677), the Court of Appeal decided that enteritis due to inhalation of sewer gas suffered by a man engaged in work in sewers was not an injury by accident. And in *Martin v. The Manchester Corporation* ([1912] 5 B.W.C.C. 259) it was also held that the contraction of scarlet fever in a fever hospital by a porter whose duty it was to clean out the mortuary and attend to the wards of a fever hospital was not an injury by accident. In *Jenkins v. Standard Colliery Co., Ltd.* ([1911] 5 B.W.C.C. 71), and *Chandler v. Great Western Railway Co.* ([1912] 5 B.W.C.C. 254) death due to blood-poisoning was also held to be outside the statute. In the former case, the decision depended entirely on the consideration of whether the physical injury had been caused in the course of the work, but in the latter the question of the actual cause of the disease was discussed. A man had injured his thumb away from his work, and the injury was the source of the infection. Both Lord Moulton and Lord Cozens-Hardy were apparently influenced in their conclusion against the claim by the fact that the dirt with which the broken surface would have been brought in contact in the course of the deceased's occupation was not from its nature a probable vehicle for germs.

Against these cases, however, there is another series which also demands attention. In *Glasgow Coal Co. v. Welsh* ([1916] S.C. (H.L.) 141) a man contracted rheumatism from standing in water which he had been directed to bale out, and this was held to be an accident within the meaning of the statute. In *Drylie v. Alloa Coal Co.* ([1913] S.C. 549), where a miner on the breakdown of the pumping machine was kept standing in water and contracted pneumonia, it was held that it was an injury due to accident within the meaning of the statute. And in *Brown v. John Watson, Ltd.* ([1915] A.C. 1), under similar circumstances, the same conclusion was reached.

It is this latter series of decisions that have received the approval of the House of Lords in the recent case of *Grant v. Kynoch* (35 T.L.R. 257) James Grant, the husband of the appellant, was a workman engaged at 25*s.* per week wages in handling and bagging artificial manures. These manures are highly impregnated with the germs known as streptococci and staphylococci. James Grant, while engaged at his work, had an abrasion on his left leg. It was not known how it was caused, and it could not be related to his employment. Infection took place at this spot, and he became ill on January 31, 1916, while engaged at his work, and died on February 16. It was not possible to state within any exact limit of time when the infection actually occurred, but the arbitrator found the following facts: that the infection which caused the illness and death was derived from poisonous germs contained in bone-dust handled in the course of his employment. He awarded compensation, and on this decision being recalled by the Court of Session the case came before the House of Lords. The question to be answered was this: Was the finding of the arbitrator justified, and did it show that the injury was due to an accident in the course of and arising out of the workman's employment? The Court, by a majority, held that the connection between the work and the disease was not, as a matter of fact, too remote, for the arbitrator had found as a fact that the infection setting up blood-poisoning was due to the impregnation of germs acquired by the man in the course of and arising out of his employment. It was an accident that the germs fell upon the deceased. It was an accident that they came in contact with the abraded surface of his skin, and from these accidental circumstances resulted the illness which ended in death. Compensation was thus ultimately awarded to the applicant, the original finding of the arbitrator being restored.

The case does not perhaps go the length of disapproving of the decision in *Martin v. Manchester Corporation* (*supra*), but it has re-opened the whole question there raised, and it has expressly overruled the procedure of the Court of Appeal in *Elke v. Hart-Dyke* (*supra*), in which the Master of the Rolls laid down that "unless the applicant can indicate the time, the day, circumstance, and place in which the accident has occurred by means of some definite event, the case cannot be brought within the general provisions of the Act, and does not entitle the workman or his dependants to compensation." In *Grant v. Kynoch* this is how the Lords of Appeal treated the principle stated by the Master of the Rolls. Lord Wrenbury said, "I think the Act is satisfied if, having regard to the nature of the particular injury alleged, the date of the occurrence of the accident is reasonably fixed so as to connect the injury with the accident"; and Lord Parmoor thus expressed himself on the point: "I am unable to draw any distinction between an accident which has been proved to have occurred at a particular day and an accident in reference to which the particular hour or day cannot be established, but which certainly is proved to have occurred within some narrow limitation of time. The question is not so much the minute particularity of the occurrence as the existence of competent evidence on which the arbitrator may find the causal connection between the accident and the injury." It is obvious that these statements of the law are much more favourable to claimants than cases of the type of *Martin v. Manchester Corporation.*

